# Transcriptomic and metabolomic analyses for providing insights into the influence of polylysine synthetase on the metabolism of *Streptomyces albulus*

**DOI:** 10.1186/s12934-022-01953-8

**Published:** 2022-10-28

**Authors:** Congcong Lian, Min Zhang, Jiaqi Mao, Yuanyu Liu, Xiuwen Wang, Linghui Kong, Qingshou Yao, Jiayang Qin

**Affiliations:** College of Pharmacy, Binzhou Medical University, Yantai, 264003 People’s Republic of China

**Keywords:** ε-poly-l-lysine, Polylysine synthetase, *Streptomyces albulus*, Transcriptomics, Metabonomics

## Abstract

ε-poly-l-lysine (ε-PL) is the main secondary metabolite of *Streptomyces albulus*, and it is widely used in the food industry. Polylysine synthetase (Pls) is the last enzyme in the ε-PL biosynthetic pathway. Our previous study revealed that Pls overexpressed in *S. albulus* CICC11022 result in the efficient production of ε-PL. In this study, a Pls gene knockout strain was initially constructed. Then, genomic, transcriptomic and metabolomic approaches were integrated to study the effects of the high expression and knockout of Pls on the gene expression and metabolite synthesis of *S. albulus*. The high expression of Pls resulted in 598 significantly differentially expressed genes (DEGs) and 425 known differential metabolites, whereas the inactivation of Pls resulted in 868 significant DEGs and 374 known differential metabolites. The expressions of 8 and 35 genes were negatively and positively associated with the Pls expression, respectively. Subsequently, the influence mechanism of the high expression and inactivation of Pls on the ε-PL biosynthetic pathway was elucidated. Twelve metabolites with 30% decreased yield in the high-expression strain of Pls but 30% increased production in the Pls knockout strain were identified. These results demonstrate the influence of Pls on the metabolism of *S. albulus*. The present work can provide the theoretical basis for improving the production capacity of ε-PL by means of metabolic engineering or developing bioactive substances derived from *S. albulus*.

**Supplementary Information:**

The online version contains supplementary material available at 10.1186/s12934-022-01953-8.

## Introduction

ε-poly-l-lysine (ε-PL), a homopoly(amino acid) consisting of 25–35 l-lysine residues, was first found in the fermentation broth of *Streptomyces albulus* [1]. ε-PL has broad spectral antimicrobial properties and a good inhibitory effect on Gram-positive bacteria, Gram-negative bacteria, yeasts, fungi, and some viruses [2, 3]. Compared with traditional chemical food preservatives (e.g., benzoic acid and sorbic acid), ε-PL has the advantages of wide antibacterial spectrum, strong antibacterial ability, high temperature resistance, good water solubility, ease of use, low dosage, and high safety [4, 5]. ε-PL has been certified by the US FDA and the European Union, and it can be safely used in food and the human body. In recent years, ε-PL has also shown broad application prospects in the fields of medicine, chemical industry, and electronic materials as nucleic acid carriers, drug carriers, and nuclear magnetic resonance contrast agents [5, 6].

*Streptomyces albulus* is the main strain for the industrial fermentative production of ε-PL. The highest reported ε-PL production was 70.3 g/L in the liquid fermentation by *S. albulus* R6 after ribosome engineering-based strain evolution and the application of acidic pH shock strategy [7]. Although the fermentation titer of ε-PL is already high, problems of long fermentation period and low product yield, among others, need to be solved. Therefore, the related network of ε-PL metabolism in *S. albulus* must be deeply studied.

The metabolic pathway of ε-PL in *S. albulus* mainly includes glycolysis, citric acid cycle, and the diaminopimelic acid (DAP) pathway. Glucose is first converted into pyruvate through the glycolytic pathway, and the latter is converted into acetyl-CoA by pyruvate dehydrogenase complex, further entering the citric acid cycle to obtain the key intermediate product oxaloacetate (OAA). Then, OAA is converted to aspartate under the catalysis of aspartate aminotransferase (Ast), and further converted to l-lysine via the DAP pathway. Finally, l-lysine is polymerized to form ε-PL under the catalysis of polylysine synthetase (Pls) [5, 8, 9].

Pls, the last enzyme of the ε-PL synthesis pathway, was first purified from *S. albulus* NBRC14147 in 2008 with a molecular weight of 130 kDa. Pls is an unusual nonribosomal peptide synthetase (NRPS) because it has adenylation and thiolation domains but lack the traditional condensation or thioesterase domain [10]. In 2010, the research group found that the length of the ε-PL peptide chain can be determined based on the Pls itself rather than using the ε-PL degrading enzyme, and a high concentration of ATP is necessary to proceed with the full enzymatic activity of Pls. The acidic pH environment during fermentation was not utilized to inhibit ε-PL-degrading enzymes, but it was necessarily applied to ensure the intracellular accumulation of ATP [11]. After four years, the researchers found that the length of the ε-PL peptide chain is affected by the linker sequences (protein) joining transmembrane domains of Pls [12].

Regarding the study of the function of Pls in the ε-PL metabolic pathway, several laboratories have attempted to express Pls gene (*pls*) by using the constitutive promoter ermE* but without success [13, 14]. Our laboratory achieved the constitutive overexpression of *pls* in *S. albulus* CICC11022 by using the strong promoter kasOp* [15], which can significantly improve the ability of the strain to produce ε-PL. This result confirms that Pls is one of the rate-limiting enzymes in the ε-PL metabolic pathway [16]. The study also found that the overexpression of *pls* negative affected the cell growth of *S. albulus* [16]. Thus far, the effect of Pls on the gene expression and metabolite synthesis of *S. albulus* is unclear.

In this study, the influence of Pls on *S. albulus* was further investigated by constructing a Pls gene knockout strain and the completion of the genome sequencing of CICC11022. These processes allowed for the determination of the effects of the overexpression and inactivation of Pls on the gene expression and metabolite synthesis of *S. albulus* via transcriptomic and metabolomic methods.

## Materials and methods

### Strains and plasmids

The bacterial strains and plasmids used in this study are listed in Table 1. The wild strain *S. albulus* CICC11022 was purchased from the China Industrial Microbial Culture Collection (CICC). The strain was first isolated from soil in Japan, and its deposit number is NBRC 14,147 at the NITE Biological Resource Center (NBRC). The *pls* gene’s high-expression strain, namely, *S. albulus* Q-PL2, was constructed previously [16]. The thermal sensitive *Escherichia coli*/*S. albulus* shuttle plasmid pKC1139 was used for gene disruption [17]. The *E. coli* ET12567/pUZ8002 was used as the plasmid donor strain for intergeneric conjugation with *S. albulus* CICC11022 [18].

**Table 1:** Strains and plasmids used in this study

Strain and plasmid	Description^a^
Strains	
* S. albulus* CICC 11022	Wild type strain, transformation host
* S. albulus* Q-PL2	*pls* gene high expressing strain harboring pSET152-pro-rbs2-pls
* S. albulus* Q-dPLS	*pls* gene knockout strain constructed in this study
* E. coli* ET12567/pUZ8002	*rec*E, *dcm*^−^, *dam*^−^, *hsd*S, Cm^r^, Tet^r^, Str^r^, Km^r^, non-methylating plasmid donor strain for intergeneric conjugation
* E. coli* Trans5α	General cloning strain
Plasmids	
pSET152-pro-rbs2-pls	Apr^r^, pSET152 carrying *kasO*p* promoter, RBS2, and *pls* gene
pKC1139	Apr^r^, temperature-sensitive shuttle vector for gene knockout
pKC1139-dpls	*pls* gene knockout plasmid based on pKC1139

^a^Cm, chloramphenicol; Tet, tetracycline; Str, streptomycin; Km, kanamycin; Apr, apramycin

### Strain culture and fermentation conditions

The MS solid medium containing 20 g/L of mannitol, 20 g/L of soybean powder, and 20 g/L of agar powder was used to culture the spores of *S. albulus* strains. The seed of *S. albulus* was cultured using the M3G medium composed of 50 g/L of glucose, 10 g/L of (NH_4_)_2_SO_4_, 5 g/L of yeast extract, 0.5 g/L of MgSO_4_·7H_2_O, 0.8 g/L of K_2_HPO_4_, 1.36 g/L of KH_2_PO_4_, 0.03 g/L of FeSO_4_·7H_2_O, and 0.04 g/L of ZnSO_4_·7H_2_O at an initial pH of 6.8. The fermentation medium for *S. albulus* was the M3G medium supplemented with 5 g/L of sodium citrate. *E. coli* strains were aerobically cultured at 37 °C by using the Luria–Bertani (LB) medium, which contained 10 g/L tryptone, 5 g/L yeast extract, and 10 g/L sodium chloride. When required, antibiotics were used at the following concentrations: 50–80 µg/mL apramycin, 25–50 µg/mL chloramphenicol, 40–50 µg/mL kanamycin, and 25 µg/mL nalidixic acid.

The cell growth and ε-PL production ability were compared by initially culturing *S. albulus* CICC11022 and the derivative strains on the MS solid medium for 5–6 days at 30 °C. Then, the spores were collected, inoculated into 50 mL of the M3G medium, and cultured at 30 °C and 220 rpm for 48 h. Finally, 5 mL of the seed culture was inoculated into 50 mL of the fermentation medium. After 72 h of cultivation at 30 °C and 220 rpm, the cell growth and the concentration of ε-PL were determined.

### Molecular manipulations

The genomic DNA was extracted using the GeneJET Genomic DNA Purification Kit (Thermo Fisher, USA). The plasmid DNA was isolated using the GeneJET Plasmid Miniprep Kit (Thermo Fisher, USA). The GeneJET Gel Extraction Kit (Thermo Fisher, USA) was used for DNA purification. Polymerase chain reaction (PCR) was performed using 2 × Phanta Max Master Mix (Vazyme, China), with the primers prepared by Sangon Biotech (Shanghai, China). The pEASY^®^-Basic Seamless Cloning and Assembly Kit (Transgen, China) was used to integrate the vector and the DNA inserts. The total RNA was extracted using RNAiso Plus (Takara, China). The cDNA was reverse-transcribed using *EasyScript*® One-Step gDNA Removal and cDNA Synthesis SuperMix (Transgen, China). The qRT-PCR reactions were performed using the QuantiNova SYBR Green PCR Kit (Qiagen, Germany) on a LightCycler 96 instrument (Roche, Germany).

### Construction of *pls* gene knockout strain

The primer pairs of dpls-up-F/dpls-up-R and dpls-down-F/dpls-down-R (Additional file 1: Table S1) were used to amplify the upstream and downstream homology arm sequences of the *pls* gene from the genome of *S. albulus* CICC11022, respectively. The PCR condition was implemented as follows: 95 °C for 15 s, 60 °C for 15 s, and 72 °C for 3 min, with 30 repeated cycles. The obtained upstream and downstream DNA fragments were integrated into the *Hind* III and *EcoR* I double-digested vector pKC1139 via Gibson assembly and transformed into *E. coli* Trans5α. The right transformants were selected via enzyme digestion, PCR, and DNA sequencing. The obtained gene knockout plasmid pKC1139-dpls was first transformed into *E. coli* ET12567/pUZ8002 and then introduced into *S. albulus* CICC11022 via intergeneric conjugation following a previously reported protocol [16]. The obtained spores were spread on MS plates containing apramycin and cultured at 37 °C for 6–8 days to obtain a single exchange strain. The single exchanged strain was subcultured at 37 °C for more than 2 generations. The obtained spores were simultaneously spread on both MS plates containing apramycin and MS plates with no resistance and then cultured at 30 °C for 6–8 days. The single colony demonstrated to be sensitive to apramycin was selected and verified via PCR using the external primers of dpls-up-F/dpls-down-R and internal primers of pls-F/pls-R of the target gene (Additional file 1: Table S1). Finally, after DNA sequencing verification, the *pls* gene knockout strain of *S. albulus* Q-dPLS was obtained.

### Genome sequencing of *S. albulus* CICC11022

The genomic DNA of *S. albulus* CICC11022 was first extracted and sheared into smaller fragments of desired size via Covaris or G-tubes methods. The Illumina PE (400 bp) library and the PacBio RS II (~ 10 K) library were constructed, sequenced using the Illumina Hiseq platform and the PacBio Sequel II platform, and assembled de novo to obtain a complete genome map for bioinformatic analysis (Majorbio Bio-Pharm Technology Co., Ltd., Shanghai, China). The sequencing data of *S. albulus* CICC11022 were deposited in the NCBI Sequence Read Archive under accession number SRR20280310 (second-generation sequencing) and SRR20644640 (third-generation sequencing).

### RNA-sequencing and transcriptomic analysis

The influence of Pls on the gene expression of *S. albulus* was investigated by isolating the total RNA of *S. albulus* CICC11022 (wild), Q-PL2 (high), and Q-dPLS (del) after 36 h of fermentation (Additional file 1: Fig. S1) and treatment with DNase I. Three biological replicates were performed. The quality and quantity of the purified RNA were determined by measuring the absorbance at 260 nm/280 nm (A_260_/A_280_) using a Nanodrop 2000 spectrophotometer. The RNA integrity was further verified using an electrophoresis apparatus and an Agilent 2100 bioanalyzer. The mRNA was enriched from the total RNA using the Ribo-Zero Magnetic Kit (Gram-Positive Bacteria) (EpiCentre, Madison, WY, USA). The libraries were constructed using TruSeq™ Stranded Total RNA Library Prep Kit reagents. The sequencing was conducted on the Illumina HiSeq 2000 platform (Majorbio Bio-Pharm Technology Co., Ltd., Shanghai, China). All sequencing data were deposited in the NCBI Sequence Read Archive under accession numbers SRR20280854 (wild), SRR20280853 (high), and SRR20280852 (del).

The clean reads were aligned to the reference genome sequence of *S. albulus* CICC11022 by using the short-sequence alignment software Bowtie 2 [19]. The expression level of each transcript was calculated by RSEM using the fragments per kilobase of exon per million mapped reads method [20]. The R statistical package software EdgeR was utilized to quantify differential gene expression [21]. The significantly differentially expressed genes (DEGs) were defined when they exhibited two or greater fold change (FC) between the samples (FC ≥ 2 or ≤ − 2; FDR < 0.05). Functional enrichment analyses were performed using Gene Ontology (GO) and Kyoto Encyclopedia of Genes and Genomes (KEGG). A Bonferroni-corrected *P*-value of ≤ 0.05 was considered to be statistically significant.

### Untargeted metabolomic analysis

The influence of Pls on the metabolites of *S. albulus* was investigated by taking cells of *S. albulus* CICC11022 (wild), Q-PL2 (high), and Q-dPLS (del) after 36 h of fermentation (Additional file 1: Fig. S1). Six biological replicates were performed. The liquid chromatography tandem mass spectrometry (LC–MS)-based metabolomic detection was performed by Majorbio Bio-Pharm Technology Co., Ltd., Shanghai, China. The analytical instrument was the UHPLC-Q Exactive HF-X system fitted with Q-Exactive quadrupole-Orbitrap mass spectrometer and equipped with a heated electrospray ionization (ESI) source (Thermo Fisher Scientific, Waltham, MA, USA), and it was used to analyze the metabolic profiling in both ESI-positive and ESI-negative ion modes. ACQUITY UPLC HSS T3 (100 mm × 2.1 mm i.d., 1.8 μm; Waters, Milford, USA) was used as the column. Mobile phase A was composed of 95% water and 5% acetonitrile (with 0.1% formic acid), while mobile phase B was composed of 47.5% acetonitrile, 47.5% isopropanol, and 5% water (containing 0.1% formic acid). The injection volume was 2 μL, and the column temperature was 40 °C. The quality control samples were prepared by mixing equal volumes of extracts from all samples. The acquired LC–MS raw data were processed on the ProgqenesisQI software (Waters Corporation, Milford, USA). Then, the software was used to search and identify characteristic peaks, and the MS and MS/MS mass spectral information was matched with the metabolic database. The MS mass error was set to be less than 10 ppm, and the metabolites were identified based on the MS matching score. The expressed metabolites with obvious differences were screened. From the selected differential metabolites, those with a variable importance plot (VIP) of > 1 and *P* of ≤ 0.05 were screened using KEGG according to OPLS-DA and UHPLC-MS/MS.

### Quantitative real-time PCR (qRT-PCR)

qRT-PCR was used to compare the expression levels of *pls* gene in the different *S. albulus* strains and verify the results of the RNA sequencing. All samples were taken after 36 h of fermentation in the same manner used for RNA sequencing. The PCR condition was implemented as follows: 95 °C for 10 s and 60 °C for 30 s in 40 repeated cycles. The RNA polymerase sigma factor (*hrdB*) was selected as the reference gene [16]. The genes and primers used for qRT-PCR are shown in Additional file 1: Table S2. The relative gene expression data were analyzed via the 2^−ΔΔCt^ method. All qRT-PCR runs were conducted with three biological and three technical replicates.

### Analytical methods for determination of growth and ε-PL production

The cell growth was measured by detecting the optical density of the samples at 600 nm using a spectrophotometer. The ε-PL concentration was determined following a previously described method [16]. All samples were measured three times. The results were plotted using the GraphPad Prism 8.3 software (GraphPad Software, USA). Statistical analysis was performed using Ordinary one-way ANOVA with Dunnett’s multiple comparisons test in GraphPad Prism 8.3.

## Results

### Effects of Pls on cell growth and ε-PL production

The *pls* gene knockout strain was first constructed to study the influence of Pls on the cell growth and ε-PL production of *S. albulus* CICC11022. The schematic diagram of the plasmid knockout vector pKC1139-dpls is shown in Additional file 1: Fig. S2A. The obtained double-exchange strain was verified by PCR. The results are shown in Additional file 1: Fig. S2B. The *pls* gene knockout strain was designated as *S. albulus* Q-dPLS and used for further investigation.

**Fig. 1:**
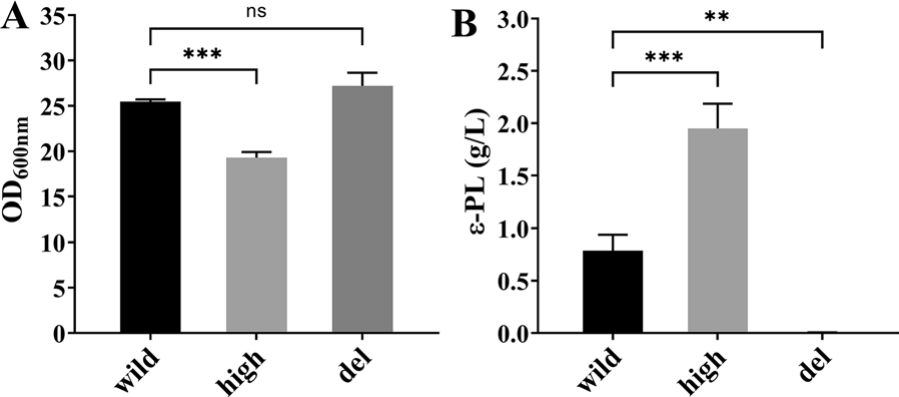
Comparison of cell growth and ε-PL production of the *pls* gene high-expression (high), knockout (del), and wild strains. **A** Comparison of the maximum OD values. **B** Comparison of the maximum ε-PL concentrations. **P < 0.01; ***P < 0.001; ns, not significant

The cell growth and ε-PL yield of *S. albulus* CICC11022 (wild), Q-PL2 (*pls* high-expression), and Q-dPLS (*pls* inactivated) were compared after 72 h of fermentation. The maximum OD value of the *pls* high-expression strain was significantly lower than those of the wild strain and *pls* gene knockout strain (Fig. 1A and Additional file 1: Fig. S1). However, the concentration of the ε-PL produced by the Pls high-expression strain was significantly higher than those of the other two strains (Fig. 1B). The *pls* gene knockout strain hardly produced any ε-PL. These results indicate that the high expression of Pls can significantly increase the production of ε-PL, but it is not conducive to the growth of the strain. Furthermore, the inactivation of the Pls causes the strain to lose the ability to produce ε-PL, but it has no significant effect on the growth of the strain.

### Genome sequencing of *S. albulus* CICC11022

**Fig. 2:**
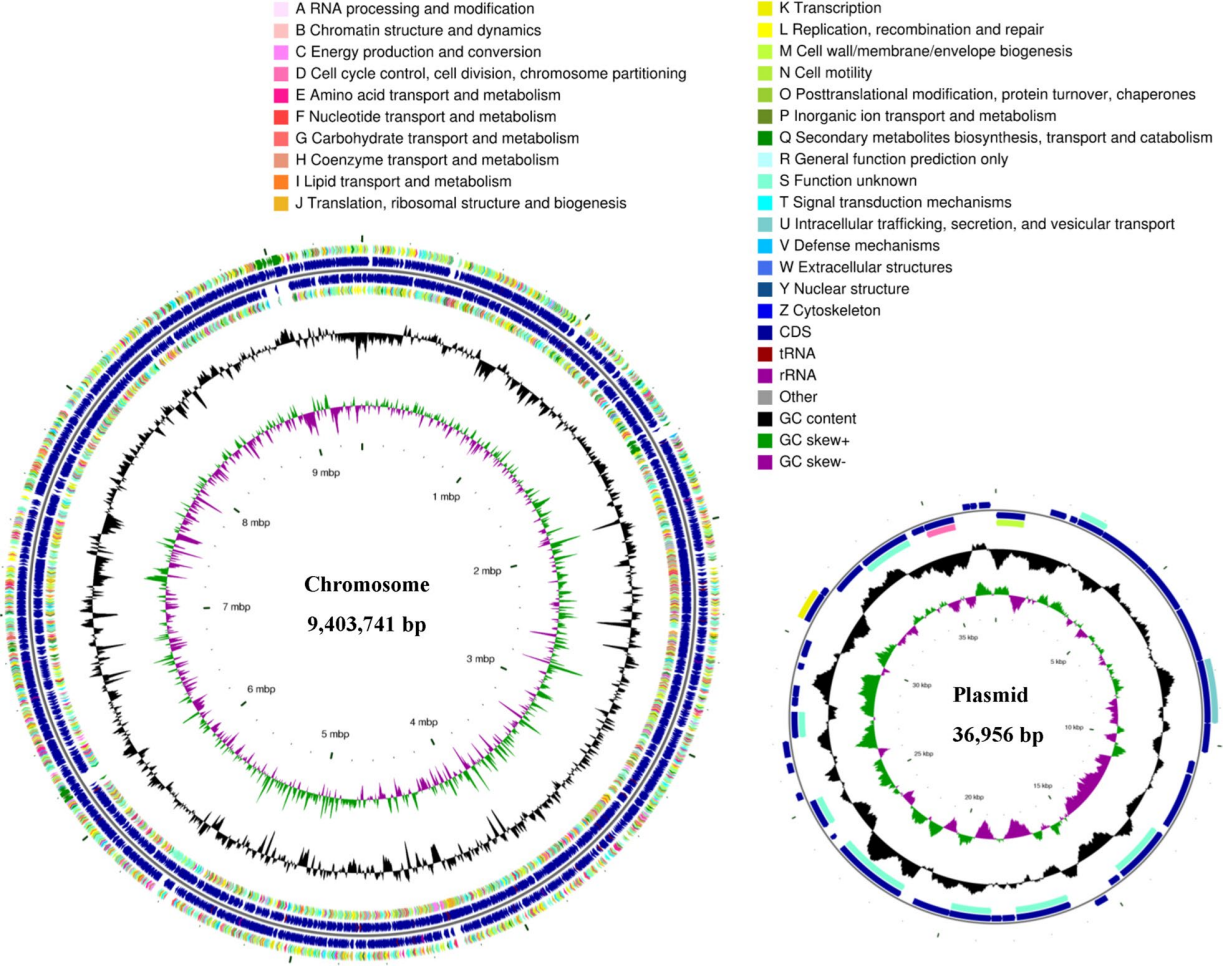
CGView genome circle map of *S. albulus* CICC11022

The total genome size of *S. albulus* CICC11022 was 9,440,697 bp, including an endogenous plasmid with a size of 36,956 bp. The genome GC content was 72.28%, and a total of 8899 CDSs, 67 tRNAs, and 21 rRNAs were predicted (Fig. 2). The results of the 16S rRNA-based evolution analysis indicate the tendency of CICC11022 to be evolutionarily close to *S. albulus* ZPM (accession number NZ_CP006871.1) among all of the studied samples (Additional file 1: Fig. S3).

### Comparative transcriptomic analysis

**Fig. 3:**
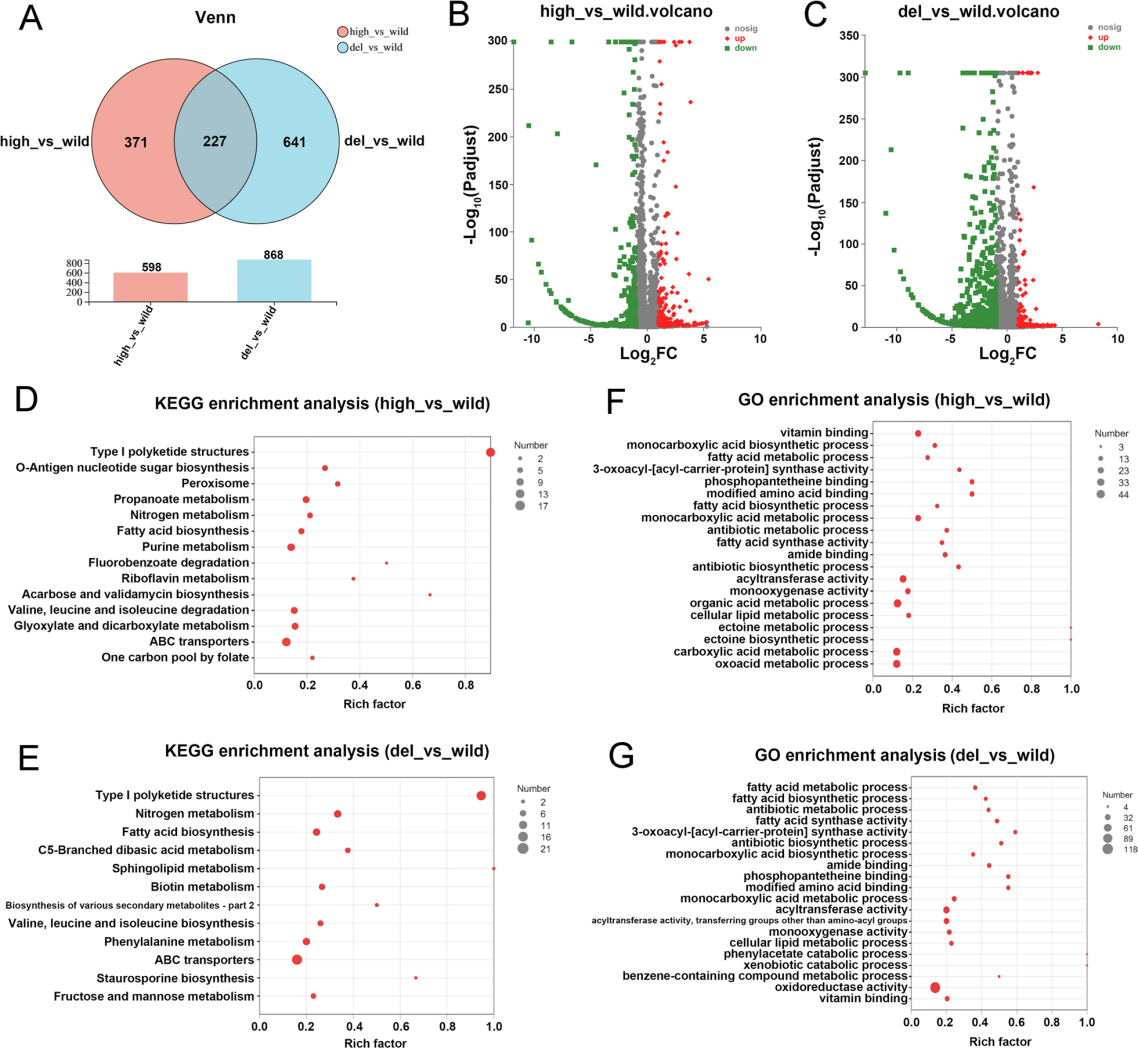
Transcriptomic comparison of the *pls* gene high-expression strain (high) and deletion strain (del) with the wild strain (wild). **A** Venn diagram of the significant DEGs. **B** Volcanic map of the significant DEGs between high and wild. **C** Volcanic map of the significant DEGs between del and wild. **D** KEGG enrich analysis between high and wild (*P* < 0.05). **E** KEGG enrich analysis between del and wild (*P* < 0.05). **F** GO enrich analysis between high and wild (*FDR* < 0.05). **G** GO enrich analysis between del and wild (*FDR* < 0.05)

To investigate the influence of Pls on the gene expression of *S. albulus*, comparative transcriptomic analysis was performed. The Venn diagram (Fig. 3A) and volcanic map of the significant DEGs between the high and wild strains (Fig. 3B) and the del and wild strains (Fig. 3C) are shown in Fig. 3. The high expression and inactivation of Pls in *S. albulus* CICC11022 resulted in 598 and 868 DEGs, respectively. The KEGG enrichment analysis showed that the top 4 most significantly regulated pathways in the *pls* high-expression strain were “type I polyketide structures” (KEGG: ko01050), “peroxisome (organelle)” (KEGG: ko04146), “O-antigen nucleotide sugar biosynthesis” (KEGG: ko00541), and “propanoate metabolism” (KEGG: ko00640) (*P* < 0.01) (Fig. 3D). The top 4 most significantly regulated pathways in the *pls* deletion strain were “type I polyketide structures” (KEGG: ko01050), “nitrogen metabolism” (KEGG: ko00910), “fatty acid biosynthesis” (KEGG: ko00061), and “C5-branched dibasic acid metabolism” (KEGG: ko00660) (*P* < 0.01) (Fig. 3E). The GO enrichment analysis showed that the top 5 most significantly regulated GO terms in the *pls* high-expression strain were “vitamin binding” (GO:0019842), “monocarboxylic acid biosynthetic process” (GO:0072330), “fatty acid metabolic process” (GO:0006631), “3-oxoacyl-[acyl-carrier-protein] synthase activity” (GO:0004315), and “phosphopantetheine binding” (GO:0031177), etc. (*FDR* < 0.001) (Fig. 3F). The top 5 most significantly regulated GO terms in the *pls* deletion strain were “fatty acid metabolic process” (GO:0006631) “fatty acid biosynthetic process” (GO:0006633), “antibiotic metabolic process” (GO:0016999), “fatty acid synthase activity” (GO:0004312), and “3-oxoacyl-[acyl-carrier-protein] synthase activity” (GO:0004315), etc. (*FDR* < 0.001) (Fig. 3G).

### Genes negatively or positively associated with the Pls expression

Among the DEGs, eight genes were significantly downregulated and upregulated in the *pls* high-expression strain and *pls* deletion strain, respectively. This finding means that the expression of these genes is negatively associated with the *pls* gene (Table 2). The expression of these genes likely has a competitive relationship with ε-PL synthesis. Among the eight genes, seven of them are adjacent to each other on the genome (Gene0445-0451). The seven genes belong to a polyketide synthase (PKS) and NRPS hybrid gene cluster. The bioinformatics analysis of the PKS–NRPS hybrid gene cluster revealed the total size of the gene cluster to be approximately 12.4 kb (Additional file 1: Fig. S4), including three PKS genes (Gene0450, Gene0448, and Gene0445), three NRPS genes (Gene0449, Gene0447 and Gene0446), and one coenzyme F420 hydrogenase (Gene0451). In the PKS gene cluster, Gene0450 is predicted to encode an enoyl reductase (ER), Gene0448 is predicted to encode keto-acyl synthase (KS), acyltransferase (AT) and keto reductase (KR), and Gene0445 is predicted to encode an acyl carrier protein (ACP). In combination, they constitute a modular type I PKS gene cluster. In the NRPS gene cluster, Gene0449 contains the adenylation (A) domain responsible for recognizing and activating the amino acid assembly unit, Gene0447 may contain an incomplete A domain, and Gene0446 contains the condensation (C) domain responsible for catalyzing the peptide bond formation. The three genes constitute an atypical NRPS gene cluster. Gene0451 is predicted to encode a coenzyme F420 hydrogenase/dehydrogenase with an unknown function. Coenzyme F420 is a flavin derivative with a chemical structure similar to that of the general flavin coenzyme FMN. Its physiological function is to act as a double-electron carrier or electron donor under low redox potential. F420 hydrogenase is a nickel iron thioflavin protein that is involved in the reduction of CO_2_ to methane with H_2_ in the methanogenic archaea. This PKS–NRPS hybrid gene cluster may be involved in the metabolism of a certain hybrid compound. At present, the synthesis mechanism of PKS–NRPS hybrid compounds is rarely studied. In the past three years, the well-studied PKS-NPR hybrid compounds only included colibrimycins [22], miharamycins [23], and acurin A [24], all of which are biologically active secondary metabolites.

**Table 2:** Genes negatively associated with the *pls* expression

Gene id	Description	High vs. wild		Del vs. wild	
		log_2_FC	*p*_adjust_	log_2_FC	*p*_adjust_
Gene0445	Hypothetical protein	− 1.23	0.00	2.79	0.00
Gene0446	Hypothetical protein	− 1.66	0.00	2.11	0.00
Gene0447	Amino acid adenylation domain-containing protein	− 2.21	0.00	2.19	0.00
Gene0448	Type I polyketide synthase	− 1.90	0.00	2.17	0.00
Gene0449	Amino acid adenylation enzyme/thioester reductase family protein	− 2.32	0.00	2.18	0.00
Gene0450	Zinc-binding oxidoreductase	− 3.42	0.00	2.24	0.00
Gene0451	Hypothetical protein	− 2.81	0.00	1.94	0.00
Gene5444	GMC family oxidoreductase	− 1.51	0.00	1.14	0.00

Among the DEGs, 35 genes were significantly upregulated and downregulated in the *pls* high-expression strain and *pls* deletion strain, respectively. This finding means that the expression of these genes is positively associated with the *pls* gene (Table 3). The expression of these genes likely has a collaborative relationship with ε-PL synthesis. Several gene clusters were identified in this section. Gene3920-3922 (*fruA*, *fruK*, *fruR2*) belong to a fructose phosphotransferase system. Gene4474-4476 (*pdhA*, *pdhB*, and *aceF*) encode the pyruvate dehydrogenase complex. Gene5032-5035 encode 3-oxoacyl-[acyl-carrier-protein] synthase II, which is a key regulator of bacterial fatty acid synthesis [25]. Gene7078-7082 encode acetolactate synthase, formyl-CoA transferase, MFS transporter and two hypothetical proteins. Other potentially valuable genes positively associated with the *pls* expression include Gene3951 (transcriptional regulator), Gene8375 (heat-shock protein Hsp20), Gene8436 (MFS transporter) and Gene8458 (transporter) because these genes may be involved in gene expression regulation and material transport.

**Table 3:** Genes positively associated with the *pls* expression

Gene id	Description	High vs. wild		Del vs. wild	
		log_2_FC	*p*_adjust_	log_2_FC	*p*_adjust_
Gene1700	Membrane protein	1.70	0.00	− 1.23	0.00
Gene3535	Hypothetical protein	1.01	0.00	− 1.45	0.00
Gene3920	PTS lactose transporter subunit IIC (*fruA*)	3.01	0.00	− 2.24	0.00
Gene3921	1-phosphofructokinase (*fruK*)	2.58	0.00	− 1.83	0.00
Gene3922	DeoR family transcriptional regulator (*fruR2*)	2.66	0.00	− 1.57	0.00
Gene3951	Transcriptional regulator	1.24	0.00	− 1.19	0.04
Gene4012	Acyl-CoA dehydrogenase	1.37	0.00	− 1.81	0.00
Gene4474	Hypothetical protein (*aceF*)	2.15	0.00	− 1.92	0.00
Gene4475	Branched-chain alpha keto acid dehydrogenase E1 subunit beta (*pdhB*)	2.25	0.00	− 1.74	0.00
Gene4476	Pyruvate dehydrogenase (acetyl-transferring) E1 component subunit alpha (*pdhA*)	1.70	0.00	− 1.39	0.00
Gene4484	Phenylacetate-CoA oxygenase subunit PaaA	1.06	0.00	− 1.54	0.00
Gene4865	Hypothetical protein	1.08	0.00	− 1.04	0.00
Gene5032	3-oxoacyl-ACP synthase (*fabF*)	1.19	0.00	− 2.51	0.00
Gene5033	Beta-ketoacyl synthase (*fabF*)	2.38	0.00	− 1.91	0.00
Gene5034	3-oxoacyl-ACP synthase (*fabF*)	1.36	0.00	− 1.35	0.00
Gene5035	Hypothetical protein	1.23	0.00	− 1.59	0.00
Gene5144	Hypothetical protein	1.49	0.00	− 1.51	0.00
Gene5145	Hypothetical protein	1.03	0.00	− 1.48	0.00
Gene5342	Hypothetical protein	1.28	0.02	− 2.13	0.03
Gene5874	Secreted hydrolase	1.29	0.00	− 2.12	0.00
Gene7029	Alpha-glucosidase	1.65	0.00	− 1.02	0.00
Gene7078	MFS transporter	2.53	0.00	− 1.72	0.00
Gene7079	Hypothetical protein	2.35	0.00	− 2.49	0.00
Gene7080	Formyl-CoA transferase	2.54	0.00	− 2.56	0.00
Gene7081	Acetolactate synthase	2.81	0.00	− 2.95	0.00
Gene7082	Hypothetical protein	2.08	0.00	− 3.30	0.00
Gene8145	Hypothetical protein	1.13	0.00	− 1.30	0.02
Gene8286	Hypothetical protein	1.07	0.02	− 1.63	0.03
Gene8330	Hypothetical protein	1.61	0.00	− 2.77	0.00
Gene8375	Heat-shock protein Hsp20	1.28	0.00	− 4.38	0.00
Gene8398	Hypothetical protein	1.03	0.03	− 1.90	0.02
Gene8431	Galactokinase	1.11	0.00	− 4.17	0.00
Gene8436	MFS transporter	1.23	0.00	− 1.45	0.03
Gene8456	DUF2306 domain-containing protein	1.40	0.02	− 2.45	0.03
Gene8458	Transporter	1.83	0.00	− 4.30	0.01

### Influence of Pls on ε-PL metabolic pathway

**Fig. 4:**
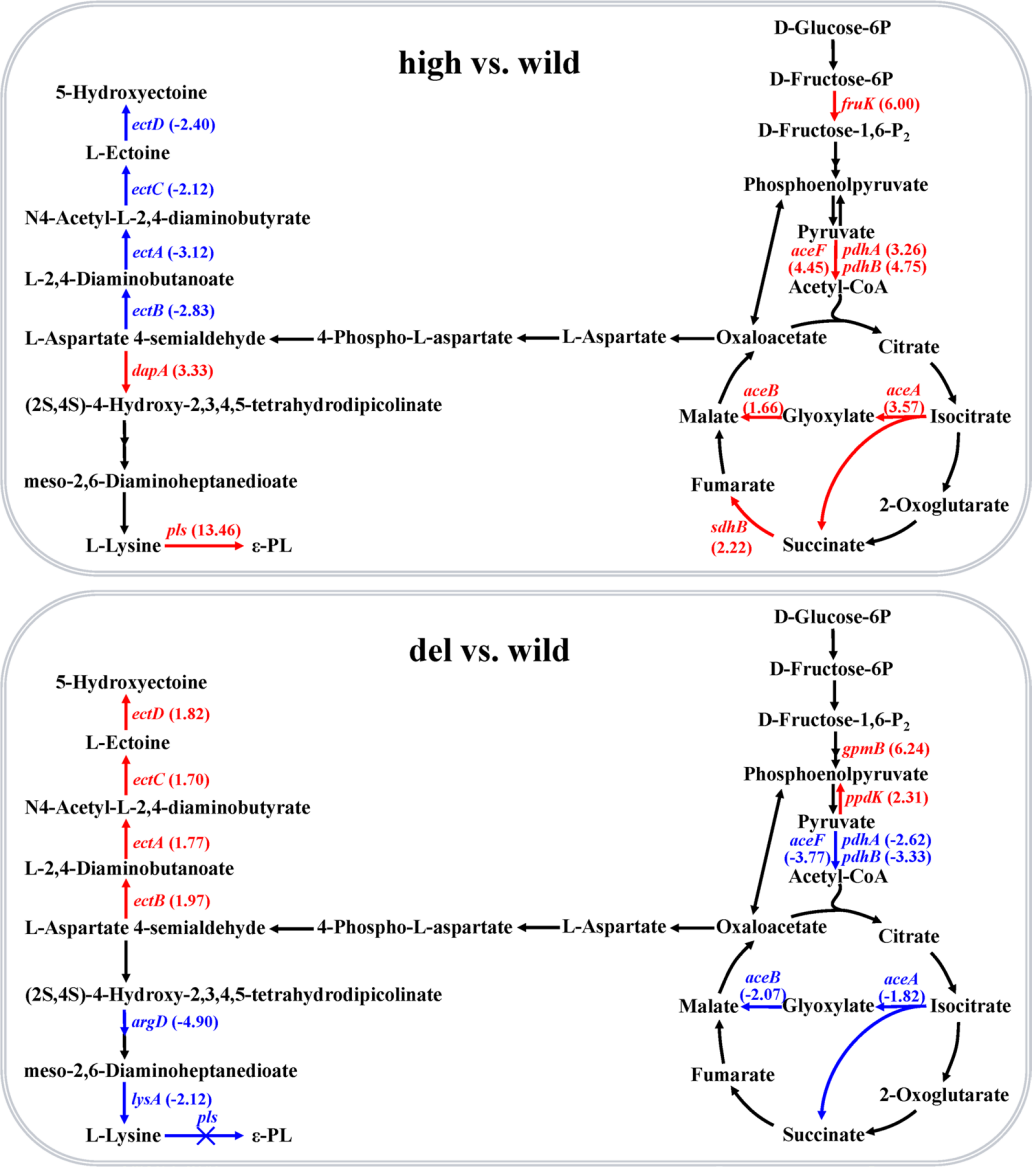
Effects of the high expression and inactivation of Pls on the expression levels of genes involved in the ε-PL metabolic pathway. The red lines, arrows and gene names represent significantly up-regulated gene expression catalyzing the step, while blue ones represent significantly down-regulated gene expression. The numbers following the gene names are the FCs in gene expression. *fruk*, phosphofructokinase; *pdhA*, pyruvate dehydrogenase (acetyl-transferring) E1 component subunit alpha; *pdhB*, pyruvate dehydrogenase E1 component subunit beta; *aceF*, pyruvate dehydrogenase complex, dihydrolipoamide acyltransferase (E2) component; *aceA*, isocitrate lyase; *aceB*, malate synthase A; *sdhB*, succinate dehydrogenase; *ectA*, L-2,4-diaminobutyrate acetyltransferase; *ectB*, diaminobutyrate-2-oxoglutarate transaminase; *ectC*, l-ectoine synthase; *ectD*, ectoine hydroxylase; *dapA*, 4-hydroxy-tetrahydrodipicolinate synthase; *pls*, polylysine synthetase; *argD*, acetylornithine aminotransferase; *lysA*, diaminopimelate decarboxylase; *gpmB*, phosphoglycerate mutase; *ppdK*, pyruvate phosphate dikinase

The metabolic pathway of ε-PL in *S. albulus* mainly includes glycolysis, the citric acid cycle, the DAP pathway, and some branch pathways [5, 8, 9]. The transcriptomic results pertaining to the influence of the high-expression and inactivation of *pls* on the gene expression involved in the ε-PL metabolic pathway are shown in Fig. 4. In the high-expression strain of Pls, the expression levels of genes encoding the pyruvate dehydrogenase complex (*pdhA*, *pdhB*, and *aceF*), isocitrate lyase (*aceA*), and malate synthase (*aceB*) were significantly upregulated, whereas the expression levels of genes encoding the l-ectoine and hydroxyectoine synthesis pathway (*ectA*, *ectB*, *ectC*, and *ectD*) were significantly downregulated (Fig. 4A). Meanwhile, in the Pls knockout strain, reverse results were obtained (Fig. 4B). The trends can be attributed to two reasons concerning the high production of ε-PL in the high-expression strains of Pls. The first reason is the activation of the glyoxylate shunt, which can synthesize OAA more efficiently with the providing of acetyl-CoA by pyruvate dehydrogenase complex. The second reason is that the metabolic pathway for aspartate semialdehyde to synthesize l-ectoine is limited, thus reducing the metabolic shunting.

### Comparative metabolomic analysis

**Fig. 5:**
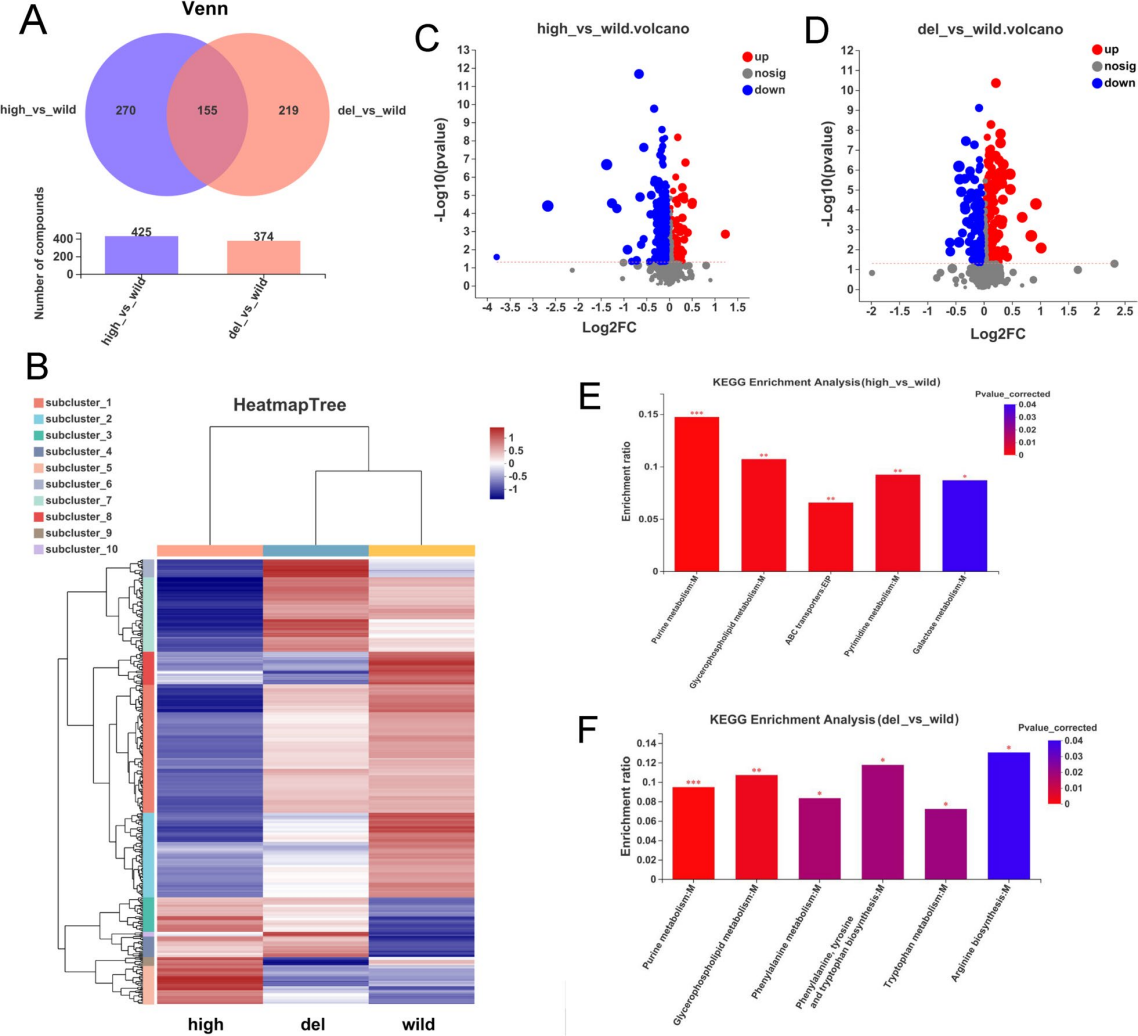
Metabolomic comparison of the *pls* gene high-expression strain (high) and deletion strain (del) with the wild strain (wild). **A** Venn diagram of the differential metabolites. **B** Clustering heat map of differential metabolites. **C** Volcanic map of differential metabolites between high and wild. **D** Volcanic map of differential metabolites between del and wild. **E** KEGG enrich analysis between high and wild. **F** KEGG enrich analysis between del and wild

To investigate the influence of Pls on the metabolites of *S. albulus*, comparative metabolomic analysis were performed. The Venn diagram (Fig. 5A), clustering heat map (Fig. 5B), and volcanic map of the differential metabolites between the high and wild strains (Fig. 5C) and between the del and wild strains (Fig. 5D) were further analyzed. The high expression and inactivation of Pls in *S. albulus* CICC11022 resulted in 425 and 374 known differential metabolites (VIP > 1), respectively. The KEGG enrichment analysis showed that the most significantly regulated pathways in the *pls* high-expression strain were “purine metabolism” (KEGG: ko00230), “glycerophospholipid metabolism” (KEGG: ko00564), “ABC transporters” (KEGG: ko02010), “pyrimidine metabolism” (KEGG: ko00240), and “galactose metabolism” (KEGG: ko00052) (*P* < 0.05) (Fig. 5E). The most significantly regulated pathways in the *pls* deletion strain were “purine metabolism” (KEGG: ko00230), “glycerophospholipid metabolism” (KEGG: ko00564), “phenylalanine metabolism” (KEGG: ko00360), “phenylalanine, tyrosine and tryptophan biosynthesis” (KEGG: ko00400), “tryptophan metabolism” (KEGG: ko00380), and “arginine biosynthesis” (KEGG: ko00220) (*P* < 0.05) (Fig. 5F).

**Fig. 6:**
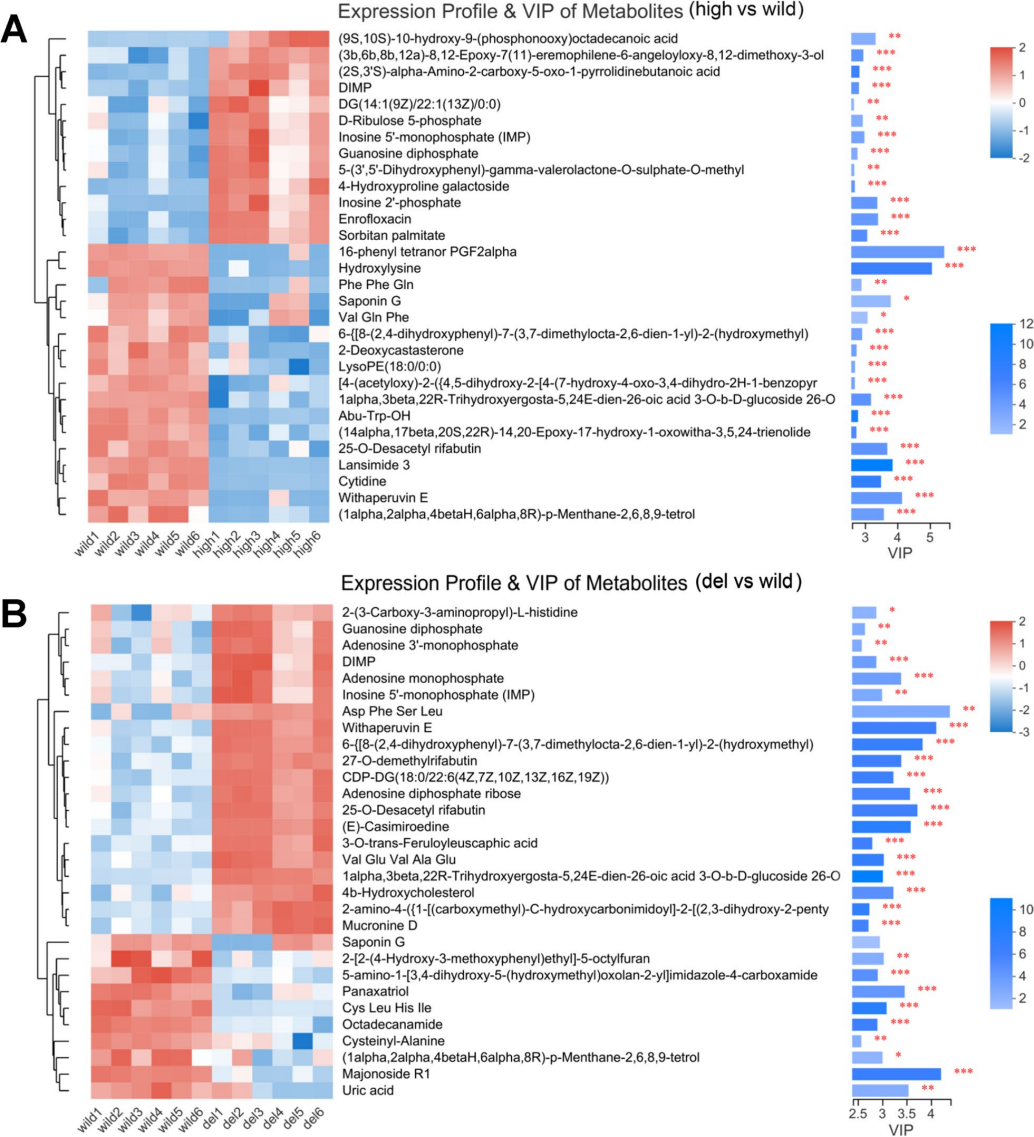
Expression profile and VIP of the identified metabolites. **A** High vs. wild. **B** Del vs. wild

The expression profile and VIP values of the identified known metabolites and total metabolites are shown in Fig. 6 and Additional file 1: Fig. S5, respectively. The differential metabolites between the high and wild groups with relatively high VIP values included 16-phenyl tetranor PGF2alpha, hydroxylysine, and withaperuvin E (VIP > 4). The amounts of all three metabolites were significantly reduced in the *pls* high-expression strain. The differential metabolites between the del and wild groups with relatively high VIP values included Asp Phe Ser Leu, withaperuvin E, and majonoside R1 (VIP > 4). The amounts of Asp Phe Ser Leu and withaperuvin E were significantly increased in the *pls* knockout strain.

**Fig. 7:**
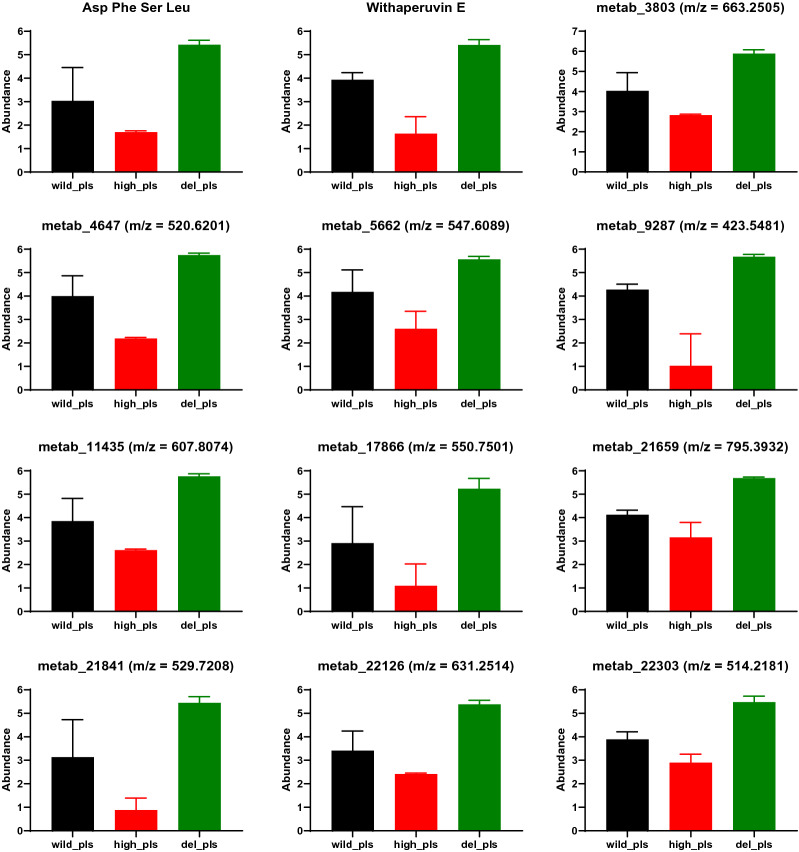
Metabolites downregulated and upregulated (> 1.3 FC) in *pls* with high-expression strain and deletion strain, respectively

After screening all differential metabolites, 12 metabolites with 30% decreased yield in the *pls* high-expression strain and 30% increased production in the *pls* knockout strain were identified (Fig. 7). These metabolites are likely to compete with ε-PL synthesis. Some of them may be biologically active, and the high-yielding strains of these metabolites can be obtained by knocking out the *pls* gene and further genetic engineering.

## Discussion

The biosynthesis of lysine in *Streptomyces* species, particularly in *S. clavuligerus*, has been widely addressed. Aspartokinase (Ask) activity in *S. clavuligerus* was found down-regulated by lysine plus threonine but up-regulated by lysine [26], and overexpression of Ask increased the concentration of cephamycin C, a downstream product of lysine [27]. Homologous expression of DAP decarboxylase (LysA) realized increased cephamycin C and tunicamycin production in *S. clavuligerus* [28]. These results indicate that Ask and LysA are two rate-limiting enzymes in the lysine biosynthesis pathway of *S. clavuligerus*. In this study, the expression of Ask was not affected by Pls overexpression and inactivation, while the expression of LysA was significantly down-regulated in the Pls inactivated strain.

The metabolic engineering method has been applied to the modification of the ε-PL synthesis pathway of *S. albulus*, and many successes have been achieved [5]. The overexpression of Pls [16], Ask [29], and dihydrodipicolinate synthase [30] and the inactivation of concomitant polyene macrolide biosynthesis [31] in *S. albulus* can increase the production of ε-PL. However, few studies have systematically modified multiple enzymes in the ε-PL pathway. One of the important reasons is the lack of an overall understanding of the ε-PL biosynthetic network of *S. albulus*.

In recent years, transcriptomic and metabolomic approaches have been successively applied in the study of the mechanism of ε-PL production. The metabolomic method and enzyme activity assays were combined to investigate the mechanism of *S. diastatochromogenes* 6#-7 for high ε-PL production; the findings demonstrated the ability of the mutant strain to enhance glucose transport and absorption capacity, increase the activities of pyruvate kinase and aspartate kinase, and decrease the activity of homoserine dehydrogenase [32]. A comparative physiology and transcriptomic analysis was also performed to elucidate the acid tolerance response of *S. albulus* M-Z18; the findings showed that acid stress could arouse the DEGs involved in transcriptional regulation, stress-response protein, transporter, cell envelope, secondary metabolite biosynthesis, DNA and RNA metabolism, and ribosome subunit [33]. Omic approaches have also been used to study the high-yielding mechanisms of mutant strain [34], pH shock strategy [35], and mixed carbon sources of glucose and glycerol [8]. However, to the best of our knowledge, omic studies in relation to high-yielding genetically engineered strains have not yet been conducted.

Pls is the last enzyme in the ε-PL biosynthesis pathway, and it is also a key rate-limiting enzyme [16]. The problems of slow fermentation rate and low product yield of ε-PL can be solved by comprehensively determining the metabolic network of *S. albulus* and then performing targeted metabolic engineering. In our opinion, the gene expression level or enzyme activity of Pls must first be improved. On this basis, genes or gene clusters that are positively and negatively coupled to ε-PL biosynthesis could be overexpressed and inactivated, respectively. Finally, the efficient production of ε-PL is expected to be achieved through the integration and optimization of genetic engineering, such as metabolic bypass knockout and biosynthesis pathway enhancement.

Besides the ability to synthesize ε-PL, *S. albulus* also has the potential to synthesize other bioactive substances, such as wuyiencin [36], toyocamycin [37], salinomycin [38], and tetramycin A and B [31]. After finishing the complete genome sequencing of *S. albulus* CICC11022, 37 secondary metabolite synthesis gene clusters were predicted by antiSMASH [39], among which 23 gene clusters had a similarity of less than 50% to known secondary metabolite synthesis gene clusters in the database (data not shown). Therefore, the inactivation of Pls may influence secondary metabolite synthesis. In this study, the PKS–NRPS hybrid gene cluster (Gene 445–451) was identified to be competitively coupled with ε-PL synthesis (Table 2). By using SeMPI 2.0 (http://sempi.pharmazie.uni-freiburg.de/index) [40], the predicted product of the hybrid PKS-NRPS gene cluster was 3(S)-Amino-4-phenyl-butan-2(S)-OL (Additional file 1: Fig. S6A) and the building blocks were 1-amino-2-phenylethyl (Additional file 1: Fig. S6B) and ethanol. 3(S)-Amino-4-phenyl-butan-2(S)-OL belongs to the class of organic compounds known as amphetamines and derivatives (https://go.drugbank.com/drugs/DB08428). The compound was not detected or not identified by metabolomic methods, while its structural analog named 3-Hydroxy-4-phenylbutan-2-one (Additional file 1: Fig. S6C) was identified and showed significantly decreased yield in the Pls high-expression strain compared with that in the wild strain (Additional file 1: Fig. S6D). Furthermore, 35 genes were identified by transcriptional analysis as having the same gene expression direction as the *pls* gene (Table 3), and they are likely to have a collaborative relationship with ε-PL synthesis. The discovery of these genes provides new targets for the genetic engineering of *S. albulus* with overexpressed Pls.

Furthermore, the comparative results of the expression levels of genes involved in the ε-PL metabolic pathway indicate that glyoxylate shunt can be activated in the *pls* high-expression strain and inactivated in the *pls* knockout strain. Previously, good results were achieved in *E. coli* by activating the glyoxylate shunt to enhance the synthesis level of aspartic acid pathway metabolites [41, 42]. However, only a few reports have linked the glyoxylate cycle to l-lysine or ε-PL in *Streptomyces*. The current study may provide new ideas for improving the production of ε-PL. Another interesting finding in this study was the ability of the l-ectoine and hydroxyectoine biosynthetic pathway to act as a competitive metabolic bypass of the ε-PL biosynthetic pathway (Fig. 4). l-Ectoine is a cyclic derivative of aspartate, while hydroxyectoine is a hydroxylated derivative of L-ectoine, and both act as osmotic protective agents in cells [43, 44]. The whole L-ectoine and hydroxyectoine biosynthetic pathway was present in *S. albulus* BCRC11814, and the expression levels of the genes were significantly upregulated in response to the increased salt concentrations [45]. Our results not only reconfirm the existence of a complete *aceABCD* gene cluster in *S. albulus* but also demonstrate the competitive relationship between the two metabolic pathways. These results not only provide ideas for the metabolic engineering of *S. albulus* to increase the production of ε-PL but also open opportunities to produce l-ectoine and hydroxyectoine by using *S. albulus*.

According to the untargeted metabolic analysis, 12 metabolites are likely to compete with ε-PL synthesis (Fig. 7). Incidentally, only two of them are known metabolites: Asp Phe Ser Leu and withaperuvin E. The function of the short peptide is unknown. Withaperuvin E, a plant-derived C28 steroidal lactone [46] belonging to a family of compounds with potential anti-inflammatory activity, is mediated by the inhibition of the tumor necrosis factor alpha (TNF-α)-induced nuclear factor kappa B (NF-κB) activity [47, 48]. Our findings suggest the existence of the synthetic gene cluster of withaperuvin E in *S. albulus* CICC11022, and the synthesis of this substance has a certain coupling relationship with Pls.

In summary, a Pls gene knockout strain was first constructed in this study. Then, genomic, transcriptomic, and metabolomic approaches were integrated to investigate the effects of the high expression and knockout of Pls on the gene expression and metabolite synthesis of *S. albulus*. Finally, the influence mechanism of Pls on the metabolism of *S. albulus* was elucidated. This study provides a theoretical basis for improving the production capacity of ε-PL by means of metabolic engineering or developing bioactive substances derived from *S. albulus*.

## Supplementary Information

**Additional file 1: Table S1.** Oligonucleotide primers used in this study. **Table S2.** Primers for qRT-PCR analysis with target gene information. **Table S3.** Expression information of selected genes for verifying RNA-seq results by qRT-PCR. **Figure S1.** Cell growth curve of the *pls* gene high-expression (high), knockout (del), and wild strains. **Figure S2.** Construction and PCR verification of the *pls* gene knockout strain of *S. albulus* CICC11022. **Figure S3.** 16S rRNA-based evolution analysis. **Figure S4.** Genomic location and function prediction of PKS–NRPS hybrid gene clusters. **Figure S5.** Expression profile and VIP of the total differential metabolites. **A**, high vs. wild; **B**, del vs. wild. **Figure S6.** Metabolite prediction and identification of the hybrid PKS-NRPS gene cluster. (A) The predicted metabolite named 3(S)-Amino-4-phenyl-butan-2(S)-OL. (B) The building block named 1-amino-2-phenylethyl. (C) The identified structural analog named 3-Hydroxy-4-phenylbutan-2-one. (D) Detection of 3-Hydroxy-4-phenylbutan-2-one by metabolomic methods.

## Data Availability

The datasets used and/or analyzed during the current study are available from the corresponding authors on reasonable request.
