# Supplementary material for: Transcriptomic and metabolomic analyses for providing insights into the influence of polylysine synthetase on the metabolism of Streptomyces albulus
Source: Microb Cell Fact. 2022 Oct 28;21:224. doi: 10.1186/s12934-022-01953-8 (PMC9617413; doi:10.1186/s12934-022-01953-8)
Supplement: Supplementary file 1 — Additional file 1: Table S1. Oligonucleotide primers used in this study. Table S2. Primers for qRT-PCR analysis with target gene information. Table S3. Expression information of selected genes for verifying RNA-seq results by qRT-PCR. Figure S1. Cell growth curve of the pls gene high-expression (high), knockout (del), and wild strains. Figure S2. Construction and PCR verification of the pls gene knockout strain of S. albulus CICC11022. Figure S3. 16S rRNA-based evolution analysis. Figure S4. Genomic location and function prediction of PKS–NRPS hybrid gene clusters. Figure S5. Expression profile and VIP of the total differential metabolites. A, high vs. wild; B, del vs. wild. Figure S6. Metabolite prediction and identification of the hybrid PKS-NRPS gene cluster. (A) The predicted metabolite named 3(S)-Amino-4-phenyl-butan-2(S)-OL. (B) The building block named 1-amino-2-phenylethyl. (C) The identified structural analog named 3-Hydroxy-4-phenylbutan-2-one. (D) Detection of 3-Hydroxy-4-phenylbutan-2-one by metabolomic methods. [file 12934_2022_1953_MOESM1_ESM.docx]

**Supplementary Material**

**Transcriptomic and Metabolomic Analyses for Providing Insights into the Influence of Polylysine Synthetase on the Metabolism of *Streptomyces albulus***

**Congcong Lian, Min Zhang, Jiaqi Mao, Yuanyu Liu, Linghui Kong, Xiuwen Wang, Qingshou Yao^*^, Jiayang Qin^*^**

College of Pharmacy, Binzhou Medical University, Yantai 264003, People's Republic of China

*Corresponding author

Email addresses:

Q.Y.: [yaoqingshou@126.com](mailto:yaoqingshou@126.com);

J.Q.: [qinjy@bzmc.edu.cn](mailto:qinjy@bzmc.edu.cn).

**Table S1** Oligonucleotide primers used in this study.

| Primers | Sequence (5'→3') | Description |
| --- | --- | --- |
| dpls-up-F | ACGACGGCCAGTGCCAAGCTTGGGACACTTGCGCAATCGT | For amplification of the upstream DNA fragment of *pls* gene |
| dpls-up-R | CTGCATCGGGCGATATGCCTCTGTTCGGTG |  |
| dpls-down-F | AGGCATATCGCCCGATGCAGTCGGCG | For amplification of the downstream DNA fragment of *pls* gene |
| dpls-down-R | CTATGACATGATTACGAATTCATGTGCTCGGACCACAGC |  |
| LS1 | GCGAGATGTGGAACACCTACGG | For qPCR detection of *pls* gene |
| LS2 | GCGAGCTGCCAGCCCTTCA |  |
| HrdB1 | CTGACCAGATTCCGCCAACCC | For qPCR detection of *hrdB* gene which is the reference gene |
| HrdB2 | GCCTCTGCGGCACTGACCAT |  |
| pls-F | ACGACAGCGTGCAGGACTGGGGGAGTTATGTCGTCGCCCCTTCTCGAATCGTCCTTC | For amplification of *pls* gene |
| pls-R | CAGGAAACAGCTATGACATGATTACGAATTCTCACGCGGCCGCACCTCCCTCCGCGCG |  |

**Table S2** The primers for qRT-PCR analysis with target gene information.

| Gene ID | Description | Primers (5΄→3΄) | Length |
| --- | --- | --- | --- |
| Gene5874 | secreted hydrolase | TGGACATGGAGTACAACCCC  TTCATCCAGTTCACCAGCCC | 73 bp |
| Gene5145 | hypothetical protein | TGGCACTGAGACACCGAATC  GTGTCGCCGCAGTCCTATC | 76 bp |
| Gene7657 | hypothetical protein | GCGAGATGTGGAACACCTACGG  GCGAGCTGCCAGCCCTTCA | 115 bp |
| Gene1172 | isocitrate lyase | CTGCTCACCGTCCTTCAACT  TGGAACTTGTAGCCCATCGC | 93 bp |
| Gene1173 | malate synthase | GCCCGTACTTCTACCTTCCG  GGAATGCCGAGCAACTCCT | 94 bp |
| Gene6380 | RNA polymerase sigma factor, *hrdB* (the reference gene) | CTGACCAGATTCCGCCAACCC  GCCTCTGCGGCACTGACCAT | 100 bp |

**Table S3** The expression information of the selected genes for verifying RNA-seq results by qRT-PCR.

| Gene ID | Description | Fold change (high vs wild) | | Fold change (del vs wild) | |
| --- | --- | --- | --- | --- | --- |
|  |  | RNA-seq | qRT-PCR | RNA-seq | qRT-PCR |
| Gene5874 | secreted hydrolase | 2.44 | 13.83 | -0.23 | -0.07 |
| Gene5145 | hypothetical protein | 2.05 | 11.88 | -0.36 | -0.22 |
| Gene7657 | hypothetical protein | 13.47 | 16.45 | -0.57 | -0.97 |
| Gene1172 | isocitrate lyase | 3.57 | 144 | -0.55 | -0.31 |
| Gene1173 | malate synthase | 1.67 | 116 | -0.48 | -0.23 |

**Fig. S1** Cell growth curve of the *pls* gene high-expression (high), knockout (del), and wild strains.


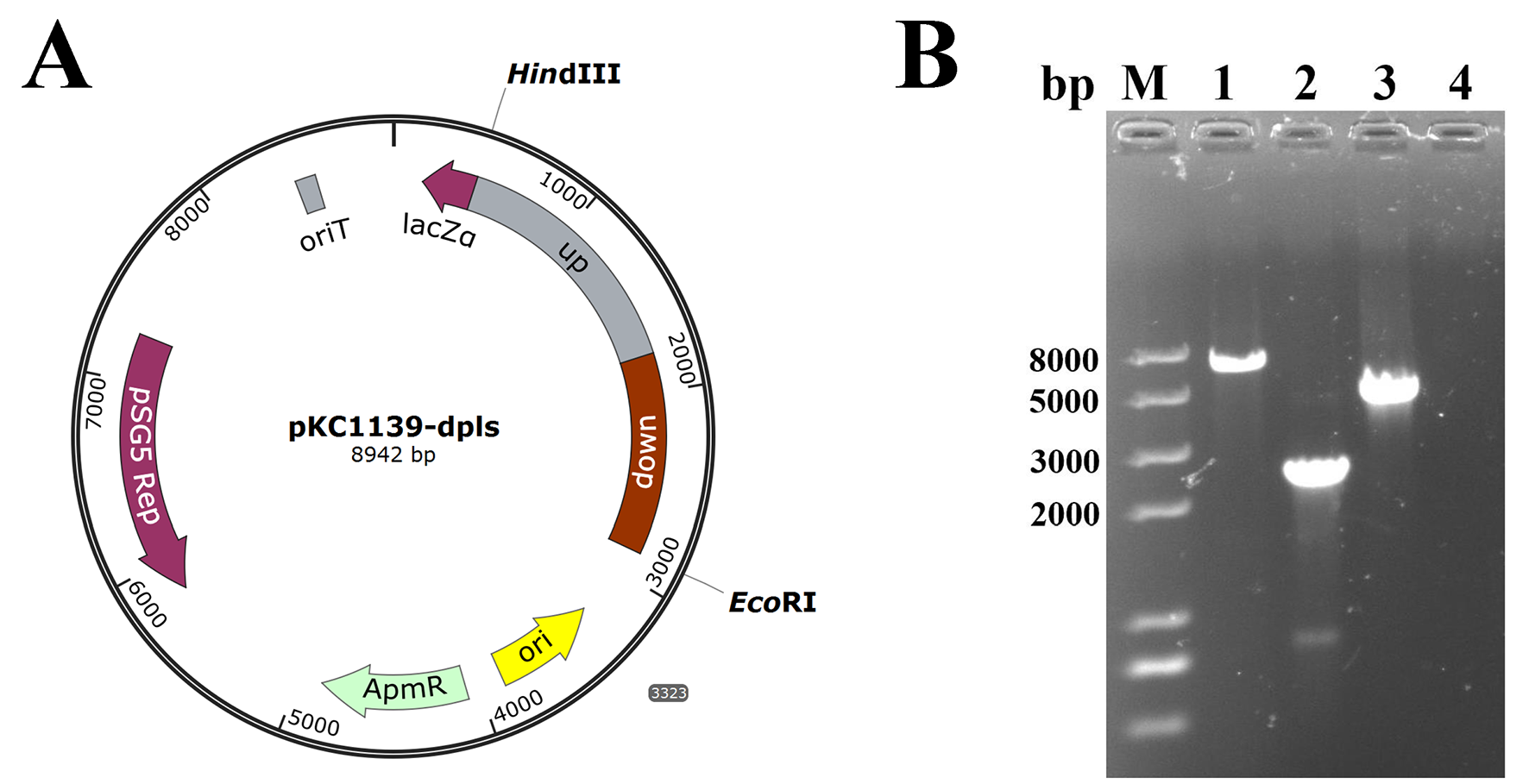


**Fig. S2** Construction and PCR verification of the *pls* gene knockout strain of *S. albulus* CICC11022. (A) Diagram of the *pls* gene knockout vector of pKC1139-dpls. (B) PCR verification using the primers outside (lanes 1 and 2) and inside (lanes 3 and 4) the *pls* gene with the genome of the wild (lanes 1 and 3) and knockout (lanes 2 and 4) strains of *S. albulus* CICC11022 as the template.

**
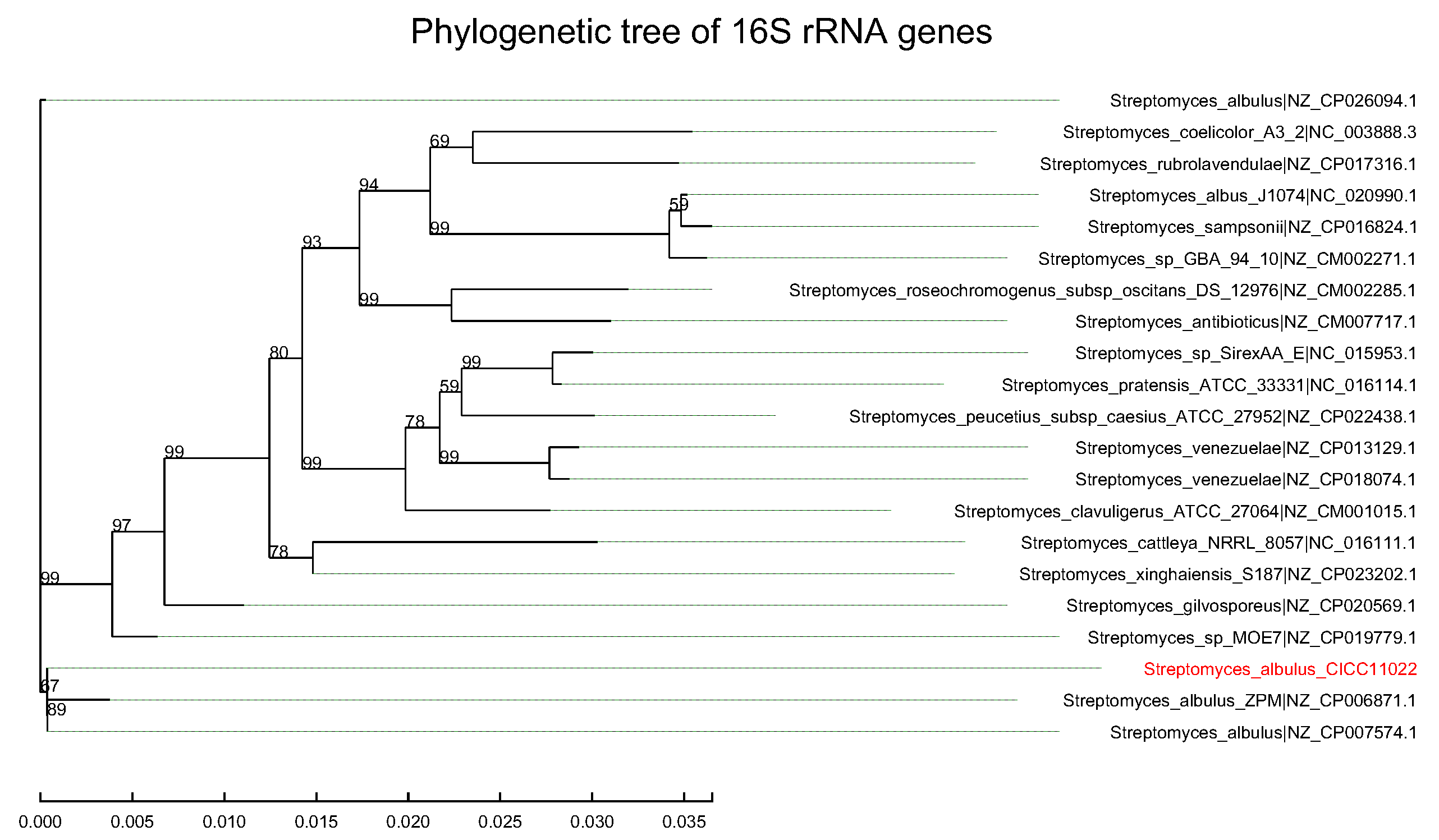
**

**Fig. S3** 16S rRNA-based evolution analysis.


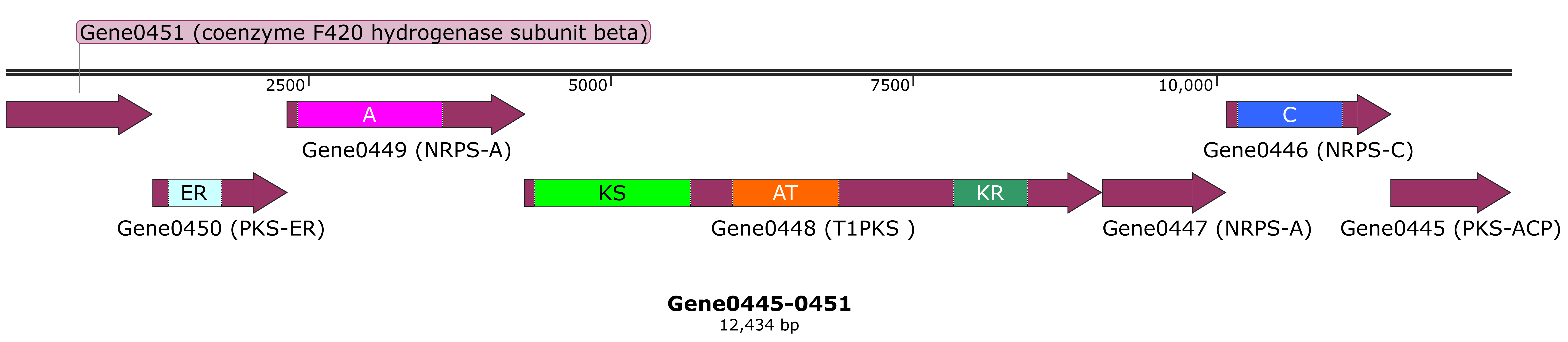


**Fig. S4** Genomic location and function prediction of PKS-NRPS hybrid gene clusters.


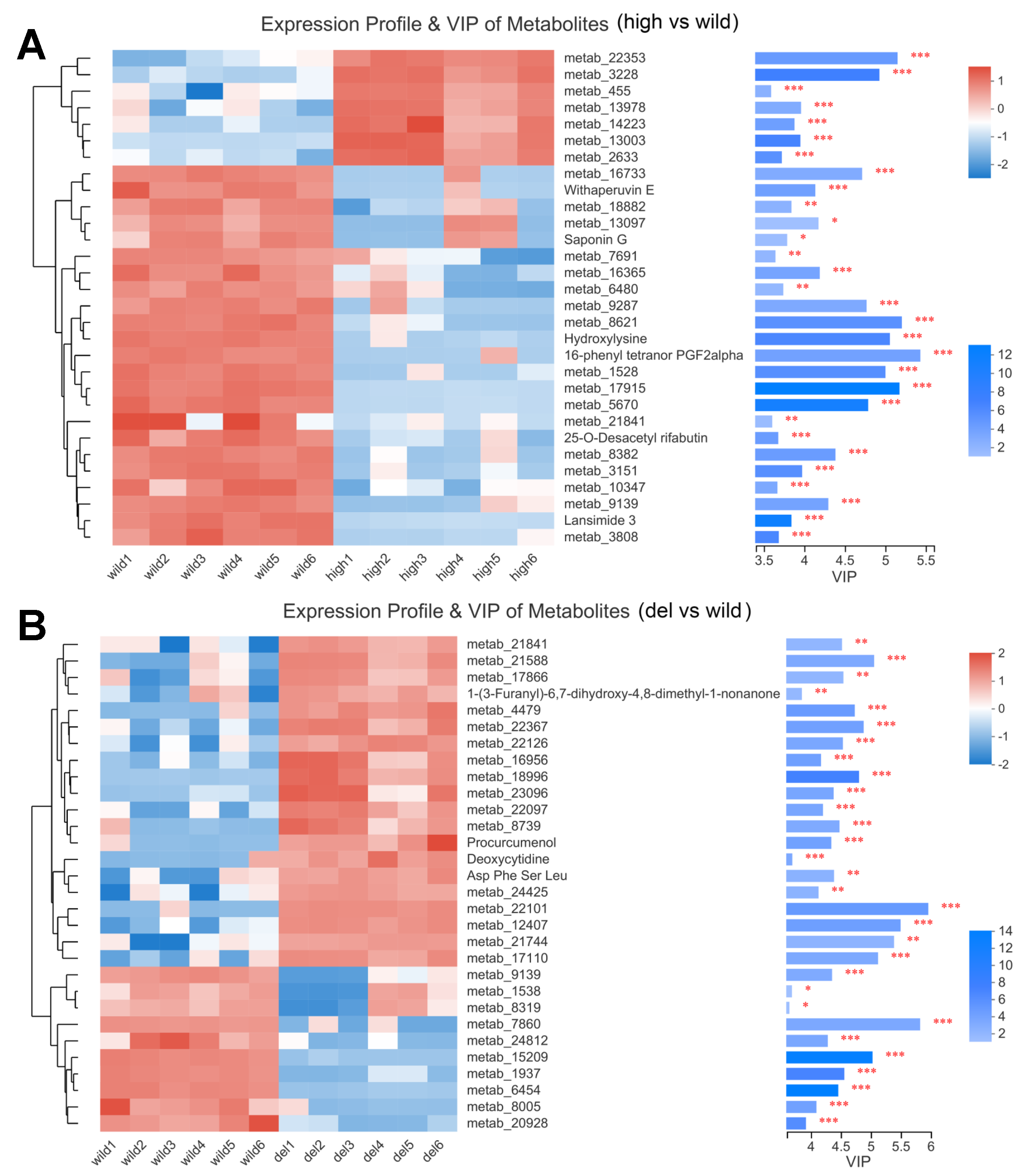


**Fig. S5** The expression profile and VIP of the total differential metabolites. (A) high vs. wild; (B) del vs. wild.


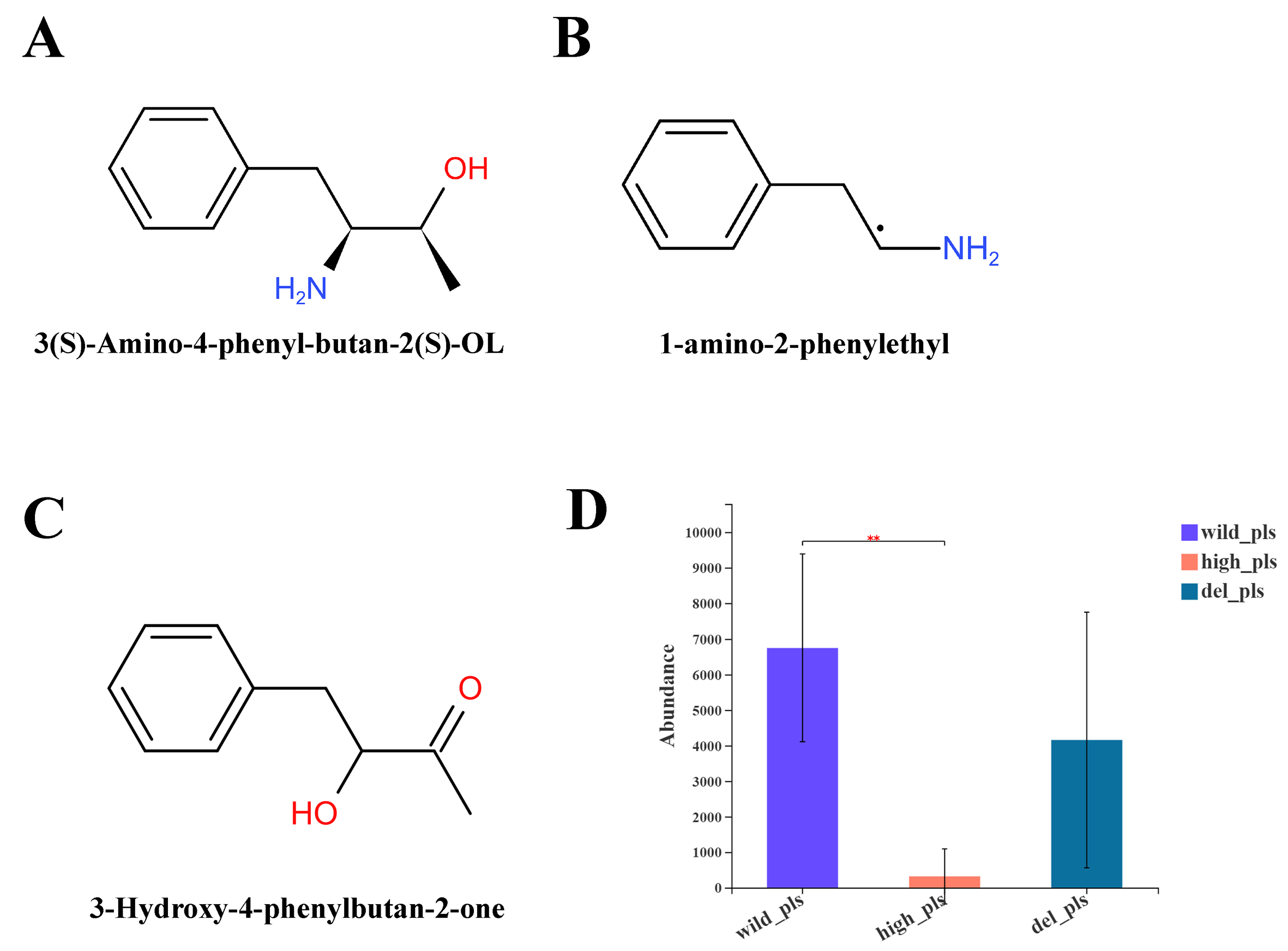


**Fig. S6** Metabolite prediction and identification of the hybrid PKS-NRPS gene cluster. (A) The predicted metabolite named 3(S)-Amino-4-phenyl-butan-2(S)-OL. (B) The building block named 1-amino-2-phenylethyl. (C) The identified structural analog named 3-Hydroxy-4-phenylbutan-2-one. (D) Detection of 3-Hydroxy-4-phenylbutan-2-one by metabolomic methods.
